# MicroRNA-197-3p Transfection: Variations in Cardiomyocyte Gene Expression with Anaesthetics Drugs in a Model of Hypoxia/Reperfusion

**DOI:** 10.3390/ph18020146

**Published:** 2025-01-23

**Authors:** Jose Luis Guerrero-Orriach, Maria Dolores Carmona-Luque, Maria Jose Rodriguez-Capitan, Guillermo Quesada-Muñoz

**Affiliations:** 1 Institute of Biomedical Research in Malaga, 29010 Malaga, Spain; guillermo.quesada.m@gmail.com; 2 Department of Anesthesiology, Virgen de la Victoria University Hospital, 29010 Malaga, Spain; la_mariose@hotmail.com; 3 Department of Pharmacology and Pediatrics, School of Medicine, University of Malaga, 29010 Malaga, Spain; 4 Maimonides Biomedical Research Institute of Cordoba, 14004 Cordoba, Spain; mdolorescarmona24@gmail.com

**Keywords:** cardioprotection, miRNA-197-3p, gene expression, human cardiomyocytes, halogenated anesthetics, sevoflurane, preconditioning, postconditioning

## Abstract

**Background:** Our research team analyzed the microRNA (miRNA)-197-3p involved in cardioprotection, and we demonstrated that the overexpression of miRNA-197-3p could be linked to a higher risk of cardiac damage. Recent research indicated that miRNA-197-3p inhibits the effector proteins of the anaesthetic preconditioning mechanism of halogenated drugs. In this scenario, we proposed to determine the role of miRNA-197-3p in cardiac injury and its effects on myocardial conditioning under halogenated exposure. **Hypothesis:** Patients having myocardial revascularization surgery have increased heart damage due to postoperative miRNA-197-3p upregulation. **Methods:** Human cardiac myocytes (HCMs) were used in an in vitro hypoxia/reperfusion (H/R) model. The miRNA-197-3p-MIMIC was transfected into the HCMs. Three H/R-induced HCM groups were performed: negative MIMIC-control transfected, MIMIC transfected, and non-transfected. Each H/R cell group was exposed to Propofol (P), Sevoflurane (S), or non-exposed. Healthy cell cultures were the control group. ELISA assays were used to assess the Akt1 and p53 cell secretion capacity, and the Next Generation Sequencing assay was used to measure the differential expression of miRNA targets. **Results**: The secretion capacity of H/R-induced HCMs transfected with the MIMIC was higher under sevoflurane exposure regarding Akt-1 cytokine (I/R + S: 0.80 ± 0.06 ng/mL; I/R + P: 0.45 ± 0.28 ng/mL; *p* > 0.05), and lower regarding p53 cytokine (I/R + S: 38.62 ± 6.93 ng/mL; I/R + P: 43.34 ± 15.20 ng/mL; *p* > 0.05) compared to propofol. In addition, a significant gene overexpression of five miRNAs, in the sevoflurane group, was linked to cardioprotection: miRNA-29-3p, 24-3p, 21-3p, 532, and miRNA-335-5p. **Conclusions:** miRNA-197-3p inhibits the cardioprotection induced by halogenated exposure and can be considered a biomarker of cardiac damage. Additional research is required to validate our findings in other clinical settings.

## 1. Introduction

It has been extensively studied and demonstrated that the selection of the hypnotic compound during the surgical process of myocardial revascularization is critical to reduce the functional and structural damage incurred during the restoration of coronary blood flow [1,2]. Several studies have analyzed the mechanisms of action triggered by halogenated drug agents administered during and after cardiac surgery, and they have demonstrated that they induce beneficial effects on the myocardium [3,4,5].

According to this research line, our group has studied this halogenated-induced cardioprotection since 2011 [4,5]. Throughout these years, we have demonstrated some of the main enzyme pathways involved at the cellular level, and some of the differences at the gene expression level occurred in the myocardium of patients exposed to halogenated compounds during the cardiac surgical process.

Moreover, we have observed the involvement of miRNAs in cardioprotection processes, and we have been able to identify the effects of micro-RNA (miRNA)-197-3p overexpression on cardiac injury and its potential role as cardiac injury biomarker. In this context, we previously showed postoperative miRNA-197-3p overexpression in patients undergoing myocardial revascularization surgery [6]. Additionally, we analyzed the effect of this miRNA in vitro. We observed a tendency towards the overexpression of this miRNA in cardiomyocytes exposed to propofol in the pre- and post-myocardial conditioning process compared to cardiomyocytes exposed to sevoflurane. This observation has been published in a recent study in which we demonstrated the critical role of miRNA-197-3p as a biomarker of cardiac damage. In this study, we observed miRNA-197-3p overexpression in cells subjected to extremely low O_2_ concentration in order to mimic the human-ischemia procedure and exposed them to non-halogenated hypnotics compounds such as propofol [7]. Moreover, in this published study, we showed that cardiomyocyte exposure to sevoflurane during myocardial conditioning induces overexpression of miRNAs related to cardioprotection, increasing the expression of cardioprotective cytokines, such as Akt-1, and decreasing the expression of cytokines related to cell damage, such as p53.

It is important to note the role of miRNA-197-3p in cancer [8,9,10,11]. Recent studies showed that the protein mechanisms of cardioprotection induced by halogenated agents and by cancer relapse share enzymatic signaling pathways, such as tyrosine kinase pathways and the Signal Transducer and Activator of Transcription (STAT) enzymatic group [12].

Sevoflurane, as a halogenated agent, exerts its protective effects on the myocardium through direct interaction with the proteins of the injury salvage kinase/survivor activating factor enhancement (RISK/SAFE) pathways group [13] and through the effector mechanism of cellular preservation in cardiomyocytes and their organelles. Thus, this agent has a special impact on mitochondrial homeostasis and the potassium channel of the mitochondrial membrane [14].

Moreover, it has been suggested that miRNA-197-3p inhibits the effector proteins involved in the anesthetic pre- and post-conditioning mechanisms induced by halogenated agents [6,7].

To continue developing new knowledge related to the effect of halogenated drugs on cardioprotection, we have performed this in vitro study as a second part of the recently published [7] to shed light on the role of miRNA-197-3p as a potential cardiac damage biomarker.

In this scenario, this in vitro study was designed to analyze the overexpression of miRNA-197-3p’s effects on cardiac injury as a diagnostic, prognostic, and therapeutic biomarker of ischemic heart disease. This study was performed in an in vitro model of Hypoxia-Reperfusion (H/R) in human cardiac myocytes (HCMs) transfected with miRNA-197-3p and exposed to sevoflurane or propofol as hypnotic drugs.

## 2. Results

### 2.1. Cytokine Quantification 

**Figure 1:**
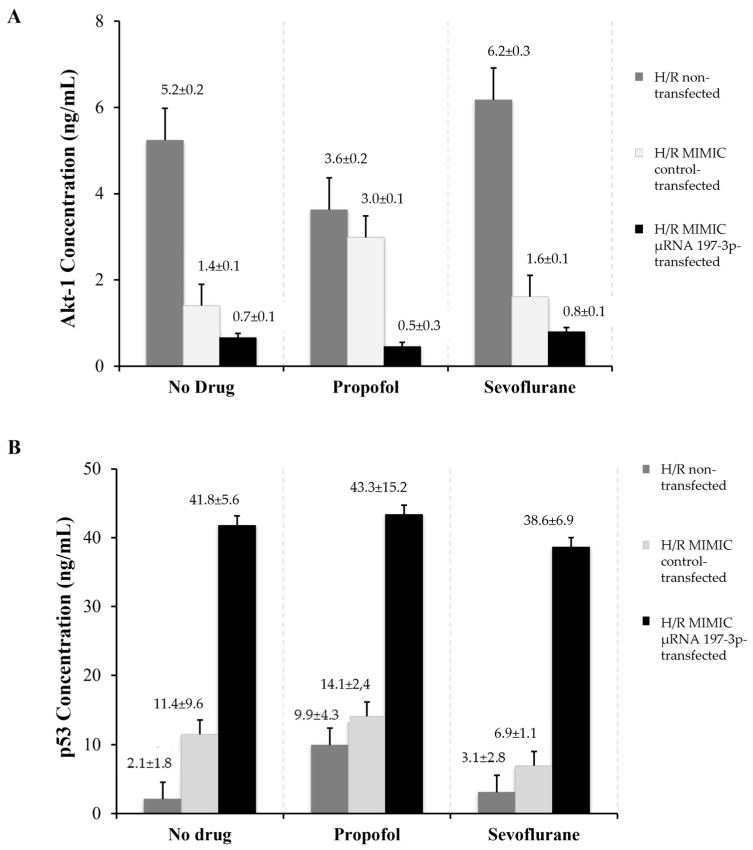
Akt-1 and p53 cytokine quantification. (**A**) Akt-1 supernatant concentration (ng/mL ± Standard Deviation (SD)) in the primary Human Cardiac Myocytes (HCMs) culture. (**B**) p53 supernatant concentration (ng/mL ± SD) in the HCMs culture. The Hypoxia/Reperfusion procedure was induced in all the HCMs analyzed and were divided into three cell types: HCM non-transfected; HCM transfected with the miRNA-197-3p MIMIC; and HCM transfected with the negative control of the miRNA-197-3pMIMIC. Each HCM type was distributed in three study groups: No drugs, without hypnotic drugs exposure; Propofol, with propofol exposure; and Sevoflurane, with sevoflurane exposure. *p*-Value non-significant: *p* > 0.05. (U-Mann Whitney statistical test).

This study revealed that, in all study groups, Akt-1 secretion was lower in all cells subjected to transfection for inducing miRNA-197-3p overexpression (Group 1, no drug: 0.66 ± 0.06 ng/mL; Group 2, Propofol: 0.45 ± 0.28 ng/mL; and Group 3, Sevoflurane: 0.80 ± 0.06 ng/mL), compared to non-transfected cells (Group 1, no drug: 5.24 ± 0.17 ng/mL; Group 2, Propofol: 3.63 ± 0.23 ng/mL; and Group 3, Sevoflurane: 6.17 ± 0.28 ng/mL). This result was confirmed by the negative transfection control included in our study (Group 1, no drug: 1.40 ± 0.14 ng/mL; Group 2, Propofol: 2.99 ± 0.08 ng/mL; and Group 3, Sevoflurane: 1.61 ± 0.13 ng/mL). These results confirmed the validity of our transfection model (Figure 1A).

Additionally, we observed that Akt-1 production was higher in miRNA-197-3p-transfected cells subjected to cardiac injury and exposed to sevoflurane, as compared to cells exposed to propofol, although the difference was not statistically significant (sevoflurane 0.80 ± 0.06 ng/mL; propofol: 0.45 ± 0.28 ng/mL; *p* > 0.05). Akt-1 secretion was lower in the cells exposed to propofol in the three cell groups (non-transfected, miRNA-197-3p-transfected, and transfected with the negative miRNA control), as compared to the other study groups (sevoflurane and non-exposure group).

The production of p53 cytokines, which are directly related to myocardial damage, was higher in miRNA-197-3p-transfected cells (Group 1, no drug: 41.78 ± 5.62 ng/mL; Group 2, Propofol: 43.34 ± 15.20 ng/mL; and Group 3, Sevoflurane: 38.62 ± 6.93 ng/mL), as compared to non-transfected cells (Group 1, no drug: 41.78 ± 5.62 ng/mL; Group 2, Propofol: 43.34 ± 15.20 ng/mL; and Group 3, Sevoflurane: 38.62 ± 6.93 ng/mL), as compared to non-transfected cells (Group 1, no drug: 2.08 ± 1.81 ng/mL; Group 2, Propofol: 9.92 ± 4.28 ng/mL; and Group 3, Sevoflurane: 3.08 ± 2.86 ng/mL) and the negative control group (Group 1, no drug: 11.49 ± 9.63 ng/mL; Group 2, Propofol: 14.09 ± 2.36 ng/mL; and Group 3, Sevoflurane: 6.92 ± 1.09 ng/mL) (Figure 1B).

In the group of HCMs transfected with miRNA-197-3p and exposed to anesthetic agents, the cells exposed to sevoflurane showed a lower capacity to produce factor p53 (38.62 ± 6.93 ng/mL), as compared to cells exposed to propofol (43.34 ± 15.20 ng/mL). However, this difference was not statistically significant (*p* > 0.05).

### 2.2. Next Generation Sequencing (NGS) Quantification

**Figure 2:**
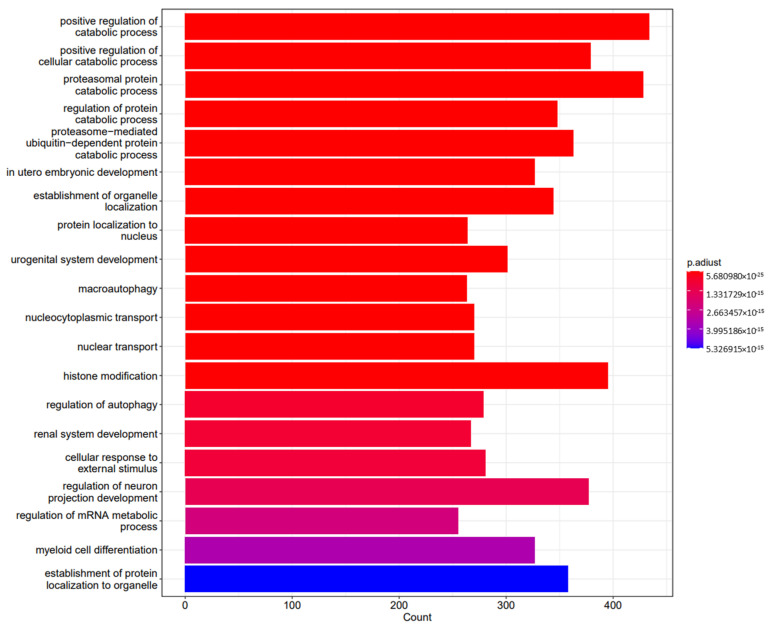
Differences between groups and crucial biological processes involved in miRNA modulation in the sevoflurane vs. propofol groups after a hypoxia–reperfusion process induced following miRNA-197-3p MIMIC transfection.

**Figure 3:**
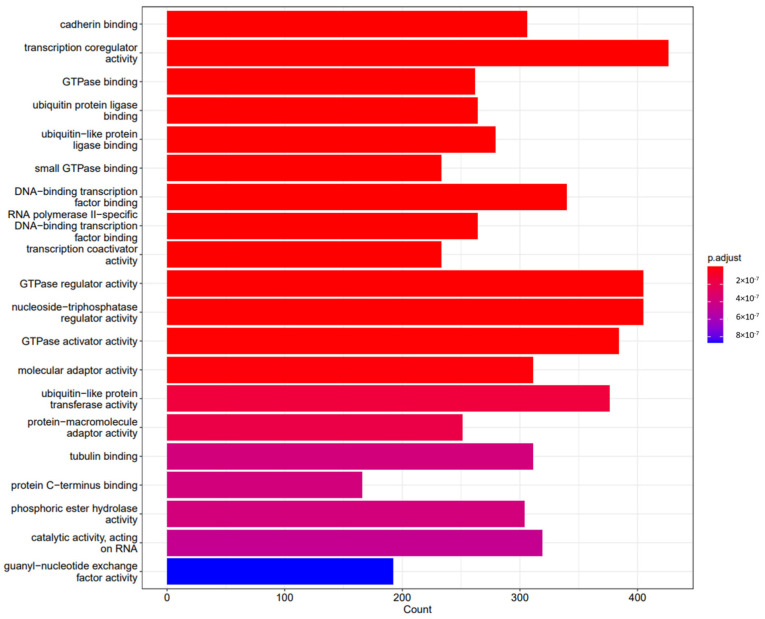
Differences between groups and crucial cellular processes involved in miRNA modulation in the sevoflurane group vs. propofol after a hypoxia–reperfusion process induced following miRNA-197-3p MIMIC transfection.

**Figure 4:**
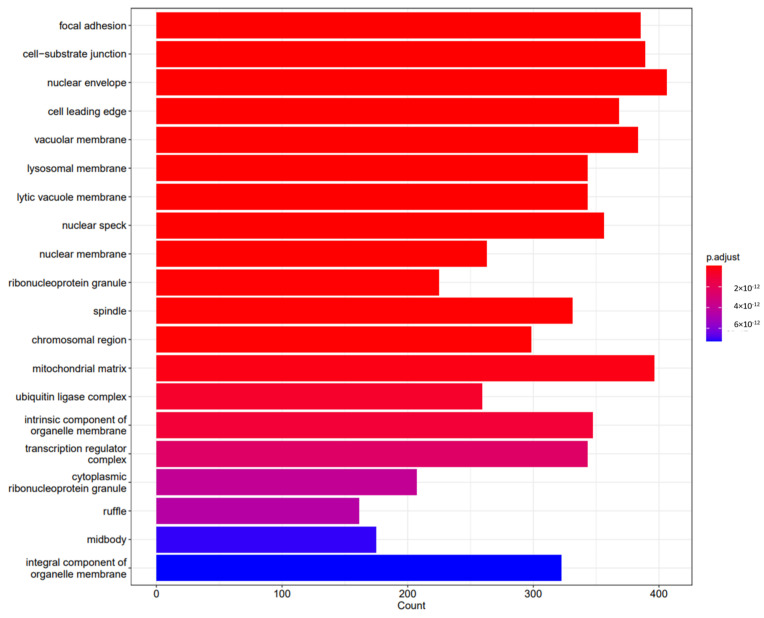
Differences between groups and crucial molecular processes involved in miRNA modulation in the sevoflurane vs. propofol groups after a hypoxia–reperfusion process induced following miRNA-197-3p MIMIC transfection.

Following functional enrichments, gene sequencing revealed how specific genes influenced different biological, cellular, and molecular processes. The results are shown in Figure 2, Figure 3 and Figure 4, respectively, and in Table 1.

## 3. Discussion

Our results demonstrated that miRNA-197-3p transfection modulated five different miRNAs in primary human cardiomyocytes exposed to propofol or sevoflurane in an in vitro H/R model. The five miRNAs overexpressed in the sevoflurane H/R cell group regarding the propofol H/R cell group were the following: miRNA-24-3p, miRNA-21-3p, miRNA-532, miRNA-29-3p, and miRNA-335-5p, which is related to cardiac protection. Moreover, our results showed that the cardiac protection effects of sevoflurane persisted even after miRNA-197-3p transfection.

All of the differentially expressed miRNAs played a significant role in the modulation of cardioprotection and myocardial pre- and post-conditioning pathways. In the two treatment groups, sevoflurane vs. propofol, miRNA-197-3p transfection and exposure to the I/R procedure induced statistically significant overexpression in two miRNAs in the sevoflurane H/R cell group compared to the propofol H/R cell group. One of them was miRNA-29-3p. The transfection of miRNA-197-3p induced variation in the expression of miRNA-29-3p. In the literature, miRNA-29-3p overexpression has been directly associated with a reduction of myocardial injury. This finding has been observed both in vitro and in patients with ischemic heart disease [15,16]. Moreover, in patients, the reduction of miRNA-29-3p concentrations correlated with the extent of the ischemic event [15]. This miRNA also operates as a modulator of systemic atheromatosis, and inflammation involved in heart failure [15,16].

The other miRNA that was significantly overexpressed was miRNA-24-3p. It has been published that the overexpression of this miRNA plays a significant role in the negative modulation of the fibroblast growth factor. Moreover, miRNA-24-3p induces the activation of a mitophagy inhibition mechanism, first described in glioma cells and subsequently in myocardial cells [17,18]. It is well known that the reduction of cardiac fibrosis is mediated by the PHB2 protein, and cardiac fibrosis is one of the main complications of ischemic heart disease. This disease impairs myocardial systolic and diastolic function and is related to heart failure in this group of patients [17].

On the other hand, significant variations were also observed in miRNA-335-5p. This miRNA is related to heart protection in ischemic injury and the reduction of intracellular calcium concentrations and apoptosis [19].

Regarding miRNA-532 overexpression, it has been documented that this miRNA has a protective effect against ischemic heart disease [20]. This effect is exerted by the regulation of fibroblast growth factors and the vascularization of the heart in patients who have experienced a cardiac ischemic event.

Additionally, miRNA-532 and miRNA-29a share the mechanism to improve myocardial remodeling and increase the potency of the effects of beta-blockers [21].

Finally, our results showed that miRNA-21-3p was down-expressed in the sevoflurane group. Previous in vitro published studies correlated propofol exposure with increased cellular senescence. The mechanisms that mediate this effect could be related to the inhibition of two enzymes with a significant role in cardiac pre- and post-conditioning, ERK½ and Akt [22]. Likewise, according to the literature, a decrease in miRNA-382 expression, similar to that observed in miRNA-21-3p in our study, was observed with differences across groups. Thus, miRNA-382 expression was proposed as a biomarker in patients with ischemic heart disease [23]. Overexpression of this miRNA has been associated with a higher risk for atrial fibrillation, a postoperative complication in patients with ischemic heart disease [24]. The incidence of this disease decreases with the use of sevoflurane as a result of its cardiac postconditioning effects [25].

Following functional enrichment, we investigated the cellular mechanisms involved in the effects of the different miRNAs’ expression. It is important to note that we found that the miRNAs identified were involved in critical cellular mechanisms, with special mention to cell cycle processes, adding to a dramatic effect on the mitogen-activated protein kinase (MAPK) and phosphatidylinositol 3′-kinase (PI3K)-Akt signaling pathways, both involved in cardiac pre- and post-conditioning, and related to an increase in cellular senescence.

The Akt-1 cytokine participates in myocardial conditioning and cardioprotection, and the p-53 cytokine is related to cellular apoptosis and cell damage processes [26]. Both cytokines’ concentrations were quantified in the cell supernatant, and we observed that H/R-induced cells transfected with miRNA-197-3p showed a variation in both cytokines in comparison to non-transfected H/R-induced cells. Our results showed that Akt-1 concentrations were higher in the sevoflurane group than in the other two groups (unexposed and propofol). These results suggest that exposure to sevoflurane favors the expression of proteins with a significant role in cardioprotection. It is important to note that before miRNA-197-3pMIMIC transfection, the Akt-1 secretion was higher in the H/R-induced cells not exposed to hypnotic drugs compared to H/R cells exposed to propofol [7].

On another note, p53 cytokine concentrations were increased in the non-transfected H/R-induced cells and in cells exposed to propofol, in comparison to the sevoflurane group. Moreover, miRNA-197-3p transfection induced an elevation of the p53 cytokine concentration in H/R cells both exposed and not exposed to hypnotic drugs compared to the non-transfected group. Nevertheless, the propofol group showed the highest p53 cytokine concentration in the cell supernatant compared to the sevoflurane group. These differences were not significant.

We can conclude that miRNA-197-3p could suppress cardiomyocyte protection mediated by exposure to halogenated agents. Moreover, we could consider sevoflurane exposure as the “best experimental conditions” according to the enzymatic measurements performed in our study. After miRNA-197-3p transfection, several variations were observed in the expression of different miRNAs in all the study groups in comparison to our previously performed in vitro study [7]. This finding suggests that miRNA-197-3p transfection inhibits the gene expression mechanisms used by sevoflurane to exert its cardioprotective effects during H/R procedures and uses “alternative pathways” to maintain cardioprotection.

Ischemia/reperfusion episodes during anesthetic procedures in cardiac surgery are quite common. In all cases where it is necessary to stop the heart to perform the intervention, cardiac arrest is induced using a series of measures aimed at protecting the myocardium. Notable among these measures are cardioplegia, local cooling, and the methods of administration of the former, which are utilized from the moment an aortic clamp is applied and coronary blood flow to the heart is ceased.

Understanding the behaviour of cardiomyocytes in situations of induced lower oxygen concentration similar to ischemia, particularly in relation to the type of anesthetic drug used, poses a challenge for clinical evaluation; this challenge is even greater when attempting to ascertain the influence of the transfection of a microRNA, as is the case in our study. The findings of our research provide a basis for the development of methods in which the cardioprotective anesthetic drug is administered during the period of cardiac arrest induced by the cardioplegia solution via the coronary ostia. This approach may lead to reduced myocardial damage and, consequently, a lower incidence of heart failure in these patients.

## 4. Materials and Methods

### 4.1. Culture and Expansion of Primary Human Cardiac Myocytes

HCMs (PromoCell GmbH, Heidelberg, Germany) were cultured and expanded from passage two to passages four or five. For this purpose, cells were seeded in culture flasks at a density of 15 × 10^3^ cells/cm^2^ in a prewarmed myocyte growth medium (PromoCell GmbH, Heidelberg, Germany), supplemented with the myocyte growth medium kit (PromoCell GmbH, Heidelberg, Germany) and 1% of penicillin/streptomycin, and incubated at 37 °C, CO_2_ 5%, and O_2_ 21% in a humidified atmosphere. The culture medium was renewed every 2 or 3 days. At 80% cell culture confluence, cells were trypsinized using the Detach Kit (PromoCell GmbH, Heidelberg, Germany), and cell counts were carried out in a Neubauer chamber, discarding dead cells using trypan blue stain.

### 4.2. Experimental Design

**Figure 5:**
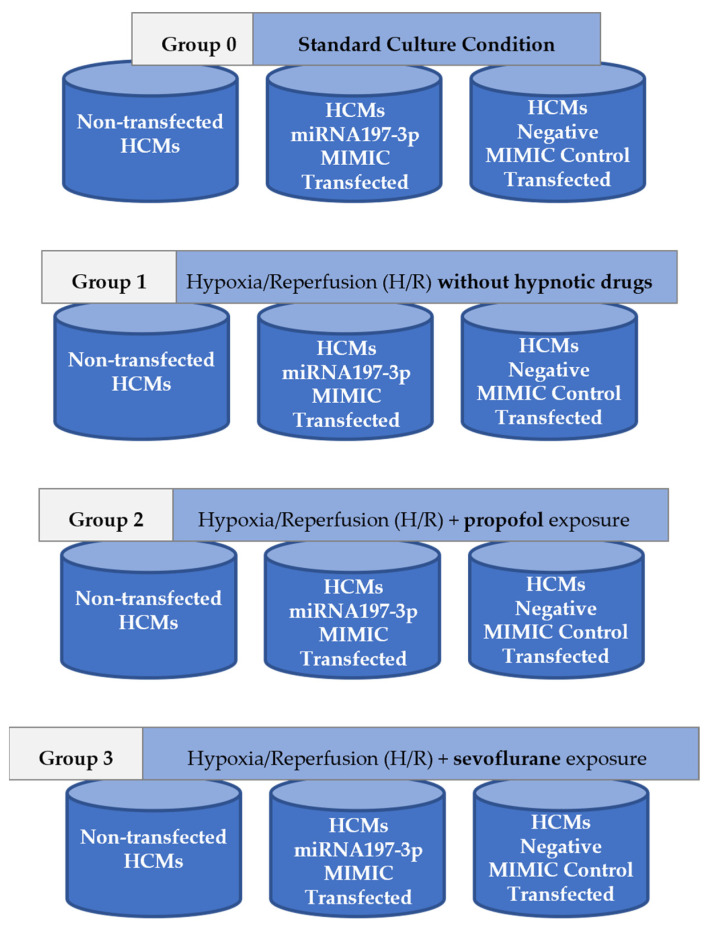
Design of the study groups.

The H/R induction procedure was performed in non-transfected HCMs, miRNA-197-3p MIMIC-transfected HCMs, and negative control MIMIC-transfected HCMs. All these cell types were included in four study groups subjected to different culture conditions: Group 0, HCMs cultured under standard conditions (n = 2); Group 1, HCMs subjected to the H/R procedure and not exposed to hypnotic drugs (n = 3); Group 2, HCMs subjected to the H/R procedure and exposed to sevoflurane as a halogenated hypnotic drug (n = 3); and Group 3, HCMs subjected to the H/R procedure and exposed to propofol as a non-halogenated hypnotic drug (n = 3) (Figure 5).

**Figure 6:**
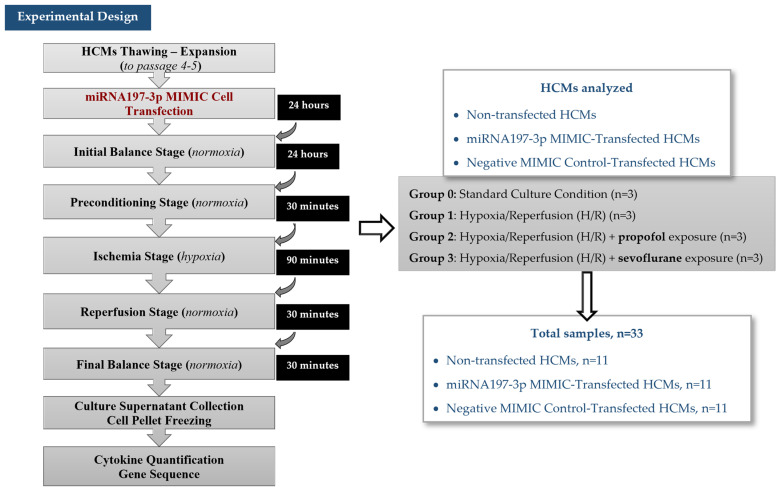
Experimental design.

To analyze the effect of the overexpression of transfected miRNA-197-3p, the differential expressions of different miRNA were quantified through a Next Generation Sequencing (NGS) assay, and the Akt-1 and p53 cytokine differential secretion was measured by performing Enzyme-Linked ImmunoSorbent Assays (ELISA) (Figure 6).

### 4.3. Hypoxia/Reperfusion Induction and Hypnotic Drug Exposure

The H/R induction was performed according to a validated in vitro model [27]. The hypnotic drugs tested in this study were sevoflurane, as a halogenated hypnotic drug, and propofol, as a non-halogenated hypnotic drug. Both anesthetics were administrated at a dose equivalent to the one used in clinical practice: 280 µM for sevoflurane (Sedana Medical AB, Uppsala, Sweden), similar to 1 minimum alveolar concentration (MAC), and 1 µM for propofol (Fresenius Kabi GmbH, Graz, Austria), equivalent to the median effective dose (ED50) applied in surgical procedures. To carry out the H/R procedure, the first step was placing the HCM culture in a complete cultured medium enriched with the corresponding anaesthetic dose, according to the study group, and incubating it for 24 h under standard culture conditions (37 °C, CO_2_ 5%, and O_2_ 21% in a humidified atmosphere). This first step to the H/R procedure was identified as the balance stage. After this, the complete medium was replaced by Hank’s Balanced Salt Solution (HBSS) supplemented with magnesium (Capricorn Scientific, Ebsdorfergrund, Germany), calcium, and cytochalasin B (5 µg/mL) (Sigma Aldrich, St. Louis, CO, USA) and enriched with the corresponding anaesthetic dose. Cells were incubated for 30 min under the same standard culture conditions. This second step was identified as the preconditioning stage. The HBSS solution was refreshed with a three times lower supplemented-HBSS volume, also enriched with the corresponding anaesthetic dose, and the cell culture was incubated at hypoxic conditions (37 °C, CO_2_ 5%, and O_2_ 1% in a humidified atmosphere) for 90 min in a hypoxia chamber (BioSpherix, Parish, NY, USA). This third step was identified as the ischemia stage. At the end of incubation under hypoxia, the reduced HBSS medium was renewed by a normal HBSS volume, enriched with anesthetics, and the cells were incubated under a standard culture for 30 min. This fourth step was identified as the reperfusion stage. Finally, the HBSS medium was replaced by a complete culture medium without anesthetic drugs, and the cells were incubated for 30 min under standard culture conditions. This last step was identified as the balance stage.

Once the ischemia procedure was completed, the culture supernatants were cryopreserved at −80 °C for the cytokine quantification, and the cells were trypsinized, washed, and cryopreserved as dry pellets to the gene sequencing assays (Figure 6).

### 4.4. Transfection Protocol

The hsa-miR-197-3p miRCURY LNA miRNA MIMIC was designed by bioNova Científica (Madrid, Spain) as miRCURY LNA miRNA MIMICs. The main aim of this design was to overexpress the target miRNA-197-3p to identify its function after cellular transfection with the designed MIMIC. For this purpose, HCMs were transfected following our transfection protocol, which was designed by our research group and validated through a previous research publication in a high-impact scientific journal [28]. The transfection protocol design is represented in Figure 5. In each of the four study groups described above, three HCM types were included: non-transfected HCMs, miRNA-197-3p MIMIC-transfected HCMs, and HCMs transfected with the negative MIMIC control. The TransIT-siQUEST^®^ transfection reagent (Mirus Bio Corporation, Madison, WI, USA) was used to transfect the HCMs. For this purpose, 5 nM of the miRNA mimic concentration was added to 3 µL of TransIT-siQUEST^®^ transfection reagent per mL of culture medium. Four hours post-transfection, the culture medium was renewed, and transfected cells were incubated under standard culture conditions for 24 h. Finally, the transfection efficiency was confirmed by optical and fluorescence microscopies using a Nikon Eclipse TE2000-S microscope (Tokyo, Japan) at a magnification of ×10.

### 4.5. Cytokine Quantification Assays

To evaluate the differential secretions of the Akt-1 and p53 cytokines regarding the miRNA-197-3p MIMIC-transfection in each study group, cell culture supernatants were collected once the process was finished and cryopreserved at −80 °C until quantification assays were performed. According to the literature, the p53 cytokine is related to cardiac damage [29], and the Akt-1 cytokine is related to cell proliferation and growth procedures [30]. For both quantifications, the Human Akt-1 (RAC-alpha serine/threonine-protein kinase) ELISA Kit (Wuhan Fine Biotech Co., Wuhan, China) and the human p53/tumour protein (p53/TP53) ELISA Kit (Cusabio, Houston, TX, USA) were used and carried out following the manufacturer’s instructions.

### 4.6. Next Generation Sequencing (NGS) Assay

The Ultra Sequencing Service and Bioinformatics Service of the Supercomputing and Bioinnovation Center of the University of Malaga (Spain) performed all analyses.

#### 4.6.1. RNA Quality for Ultra Sequencing

RNA was isolated and the miRNA quality was analyzed by lab-on-a-chip electrophoresis using the Bioanalyzer 2100 kit (Agilent, Santa Clara, CA, USA). The samples’ quantification was carried out in a Qubit fluorimeter (Life Technologies, Carlsbad, CA, USA). All the aliquots were cryopreserved at −80 °C until their processing.

#### 4.6.2. miRNA-Seq and Messenger RNA (mRNA)-Seq

The libraries were created using the NEBNext Small RNA sample preparation (New England BioLabs, Ipswich, MA, USA) for miRNA-seq according to the manufacturer’s instructions.

For mRNA-se, the Poly-A-enriched strand-specific libraries were created using the TruSeq Stranded mRNA sample preparation (Illumina, San Diego, CA, USA) according to the manufacturer’s instructions. The quality and yield of the created libraries were assessed using a Qubit 2.0 Fluorometer (Life Technologies, Carlsbad, CA, USA) and an Agilent 2100 Bioanalyzer (Agilent Technologies, Santa Clara, CA, USA). The library pools were sequenced on a Nextseq550 instrument (Illumina) according to the manufacturer’s instructions.

For miRNA-seq, simple 1 × 75 pb miRNA sequencing was performed for a minimum of 1–10 million reads per sample, and for mRNA-seq, 2 × 75 pb sequencing was performed for a minimum of 15–30 million reads per sample.

### 4.7. Analysis Data: Bioinformatics and Statistics

The analysis of miRNA gene expression data was performed through RNA-seq technology. For this purpose, a bioinformatic analysis was developed to identify differential expression between the different study groups. The analysis process involved several stages. In brief, a software developed in the Andalusian Bioinformatics Platform (PAB) called SeqTrimNext (version 2.0.68.) was used. Moreover, to identify the alignment of the readings to the genomic reference, the latest version of the human genome (hg38) was used. The level of expression was reported in the Readings Mapped Per Million (RPKM) format. To obtain the genes with differential expression, the Tuxedo tools were used, which included Cufflinks, Cuffmerge, and Cuffdiff, tools specially designed to enhance differential expression calculations from RNA-Seq data. This process involved the pre-normalization of the expression data and the comparison of the data groups. Finally, the results were included in a differentially expressed genes list of miRNAs that met the stability among the population, the minimum level of expression, and the statistical significance.

The statistical analyses derived from the other results were performed using the usual descriptive statistical analysis for non-parametric data (n < 30). The Mann–Whitney U test was used for non-parametric data comparison between two groups, and the Kruskal–Wallis test was applied to the comparisons of data between all the study groups. Statistical significance was accepted when the *p*-Value was ≤0.05. All the statistical analyzes were performed using the PASW Statistic 18 (IBM SPSS, Chicago, IL, USA) software. The results were expressed as mean (M) ± standard deviation (SD) unless otherwise stated.

## 5. Conclusions

In this study, we analyzed the effects of the overexpression of miRNA-197-3p in an in vitro model of H/R in human cardiac myocytes exposed to hypnotic drugs, such as sevoflurane as a halogenated drug, and propofol as non-halogenated drug, in order to mimic the Ischemia/Reperfusion human procedure. In the light of the results obtained, we can conclude that miRNA-197-3p overexpression inhibits the gene expression of cardioprotective factors induced by halogenated exposure. However, halogenated drugs are able to increase these effects by developing “alternative pathways” to maintain cardioprotection. In this scenario, miRNA-197-3p could be considered a biomarker of cardiac injury.

Further studies are needed to confirm our results in different clinical scenarios.

## Figures and Tables

**Table 1:** RNA sequencing results. Small RNAs and miRNAs that experienced variations before and after miRNA-197-3p transfection in hypoxia–reperfusion induction in the HCM group exposed to sevoflurane vs. propofol.

Genes	Statistically Significant Difference (*p*-Value)
*5_8S_rRNA_8*	0.001155384
*hsa-miR-197-3p*	0.001155384
*hsa-let-7c-5p*	0.001155384
*hsa-let-7b-5p*	0.001155384
*hsa-miR-323b-3p*	0.001155384
*hsa-piR-23511*	0.001155384
*hsa-piR-33031*	0.001155384
*ACA18*	0.001155384
*hsa-piR-33185*	0.001155384
*tRNA-Met-CAT-2-1*	0.001155384
*ACA20*	0.001155384
*hsa-piR-31082*	0.001155384
*ACA2b*	0.001155384
*tRNA-Val-AAC-5-1*	0.001451398
*U23*	0.001451398
*hsa-let-7f-5p*	0.001867351
*hsa-miR-23b-5p*	0.001867351
*hsa-piR-32990*	0.001867351
*hsa-piR-33005*	0.002369099
*U79*	0.002369099
*hsa-let-7d-5p*	0.002369099
*hsa-miR-193a-5p*	0.002369099
*hsa-miR-654-5p*	0.003025987
*hsa-piR-33197*	0.003025987
*hsa-miR-758-3p*	0.003025987
*hsa-miR-423-5p*	0.003025987
*hsa-piR-28727*	0.003025987
*hsa-piR-3440*	0.003025987
*hsa-miR-98-5p*	0.003025987
*ACA11*	0.003025987
*hsa-miR-1307-3p*	0.003025987
*tRNA-Gly-CCC-2-2*	0.003025987
*tRNA-Pro-AGG-2-6*	0.003025987
*tRNA-Val-CAC-chr1-134*	0.003025987
*hsa-piR-33123*	0.003906234
*hsa-miR-432-5p*	0.003906234
*hsa-piR-29204*	0.003906234
*tRNA-Gly-CCC-chr1-135*	0.003906234
*hsa-piR-33155*	0.003906234
*hsa-piR-22236*	0.004996605
*tRNA-Pro-AGG-2-4*	0.004996605
*ACA26*	0.004996605
*tRNA-Arg-CCT-4-1*	0.004996605
*hsa-piR-33165*	0.004996605
*hsa-piR-33057*	0.004996605
*ACA9*	0.004996605
*hsa-piR-32298*	0.004996605
*hsa-piR-33115*	0.004996605
*U65*	0.004996605
*hsa-miR-370-3p*	0.004996605

## Data Availability

The original contributions presented in this study are included in the article. Further inquiries can be directed to the corresponding author.
